# Post-Carotid Artery Stenting Hyperperfusion Syndrome in a Hypotensive Patient: Case Report and Systematic Review of Literature

**DOI:** 10.3390/life14111472

**Published:** 2024-11-12

**Authors:** Matija Zupan, Matej Perovnik, Janja Pretnar Oblak, Senta Frol

**Affiliations:** 1 Department of Vascular Neurology, University Medical Centre Ljubljana, Zaloška cesta 2, SI-1000 Ljubljana, Slovenia; matija.zupan@kclj.si (M.Z.); janja.pretnar@kclj.si (J.P.O.); senta.frol@kclj.si (S.F.); 2 Faculty of Medicine, University of Ljubljana, Vrazov trg 2, SI-1000 Ljubljana, Slovenia

**Keywords:** carotid artery stenting, cerebral autoregulation, cerebral hyperperfusion syndrome, endothelial dysfunction, pathophysiology

## Abstract

Cerebral hyperperfusion syndrome (CHS) is a serious post-procedural complication of carotid artery stenting (CAS). The pathophysiological mechanisms of CHS in the absence of arterial hypertension (AH) remain only partially understood. We performed a systematic literature search of the PubMed database using the terms »cerebral hyperperfusion syndrome«, »hypotension«, »hyperperfusion«, »stroke«, »intracranial hemorrhages«, »risk factors«, »carotid revascularization«, »carotid stenting«, »carotid endarterectomy«, »blood-brain barrier«, »endothelium«, »contrast encephalopathy«, and combinations. We present a case of a normotensive female patient who developed CHS post-CAS for symptomatic carotid stenosis while being hypotensive with complete recovery. We identified 393 papers, among which 65 were deemed relevant to the topic. The weighted average prevalence of CHS after CAS is 1.2% [0.0–37.7%] with that of intracranial hemorrhage (ICH) being 0.51% [0–9.3%]. Recently symptomatic carotid stenosis or contralateral carotid revascularization, urgent intervention, acute carotid occlusion, contralateral ≥70% stenosis, and the presence of leptomeningeal collaterals were associated with CHS. A prolonged hemodynamic instability after CAS conveys a higher risk for CHS. However, none of the articles mentioned isolated hypotension as a risk factor for CHS. Whereas mortality after ICH post-CAS ranges from 40 to 75%, in the absence of ICH, CHS generally carries a good prognosis. AH is not obligatory in CHS development. Even though impaired cerebral autoregulation and post-revascularization changes in cerebral hemodynamics seem to play a pivotal role in CHS pathophysiology, our case highlights the complexity of CHS, involving factors like endothelial dysfunction and sudden reperfusion. Further research is needed to refine diagnostic and management approaches for this condition.

## 1. Introduction

Cerebral hyperperfusion syndrome (CHS) is a serious post-procedural complication of carotid artery stenting (CAS) that can result in severe disability and death [1]. The most common clinical presentation is a severe headache, whereas less common and more severe symptoms include focal neurological deficits, encephalopathy, and seizures [2]. The pathophysiological mechanisms of CHS remain only partially understood. Impaired cerebral autoregulation and post-revascularization changes in cerebral hemodynamics are believed to be the main mechanisms involved in the development of the syndrome [2]. CHS has been reported more frequently after CAS than after carotid endarterectomy (CEA) and it is believed that contrast agent-mediated disruption of the blood-brain barrier (BBB) may also potentiate these mechanisms [3]. Most studies describe CHS occurring more frequently in symptomatic patients, who have contralateral carotid stenosis/occlusion, poor primary collaterals, microvascular cerebral disease, female sex, and chronic kidney disease. Preexisting long-standing arterial hypertension (AH) as well as post-procedural AH have been identified as the most important risk factors [4]. A large meta-analysis across 33 studies, concerning more than 8000 CAS patients, yielded a pooled CHS risk of 4.6%, with an average time from a procedure to symptoms of 12 h [5]. Interestingly, symptomatic status was associated with a lower risk of CHS [5]. 

Here, we present a normotensive patient who developed CHS post-CAS for symptomatic moderate carotid stenosis while being hypotensive. We provide a review of the relevant studies of CHS, focusing on the pathophysiological mechanisms involved in the syndrome in the absence of AH.

## 2. Case Report

**Figure 1:**
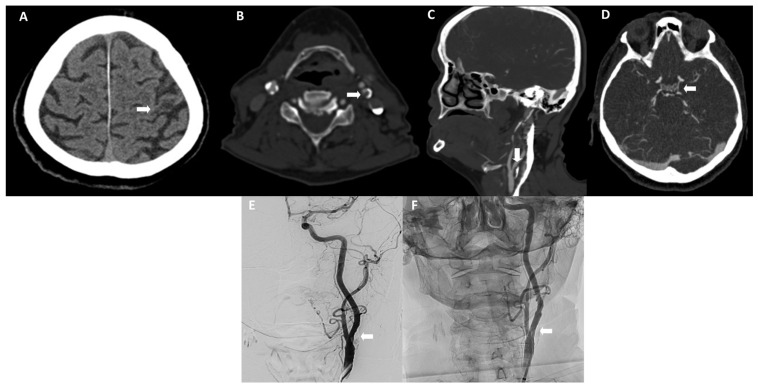
Imaging before neurological worsening. (**A**) A CT pre-procedure showing postischemic encephalomalacia in the left frontal region (arrow). (**B**,**C**) A CT angiography (CTA) pre-procedure showing a hemodynamically significant left internal carotid artery (ICA) stenosis of 80–90% (arrows). (**D**) A CTA pre-procedure showing a patent left posterior communicating artery. (**E**) A DSA showing a 50% left ICA stenosis (arrow). (**F**) The DSA showed a patent stent without residual stenosis at the end of the procedure (arrow).

A 67-year-old right-handed Caucasian female patient, a long-term smoker with a known chronic obstructive pulmonary disease (COPD), was admitted for elective CAS of the left internal carotid artery (ICA). The stenosis was revealed by diagnostic imaging, which was performed due to a month-long occipital headache and reduced coordination. She had never exhibited elevated blood pressure (BP). The neurological examination was unremarkable; the National Institute of Health Stroke Scale score (NIHSS) was 0. A non-contrast head computed tomography (CT) scan revealed a postischemic encephalomalacia in the left frontal lobe (Figure 1A). A Duplex ultrasound (DUS) and later CT angiography have shown an 80–90% stenosis of the left ICA due to a heterogeneous, marginally calcified plaque (Figure 1B,C). Other precerebral, as well as intracranial arteries, exhibited only non-obstructive atherosclerosis. The left posterior communicating artery (PCoA) was clearly visible (Figure 1D). Due to the potentially symptomatic and hemodynamically significant stenosis of the left ICA, revascularization with CAS was performed six months after the initial clinical complaint. 

On admission, the patient had no neurological deficits (NIHSS 0); she was normotensive (124/70 mmHg). The electrocardiogram was normal with sinus rhythm and 67 beats per minute. Digital subtraction angiography (DSA) of the left ICA revealed only a 50% stenosis (Figure 1E). Due to the potentially symptomatic stenosis, CAS was nonetheless performed. A bare metal carotid stent (CGUARD RX^®^ STRAIGHT 6F/0.14) measuring 8 × 30 mm was deployed and additionally dilated with a balloon (VIATRAC^®^ 14 PLUS 5F) measuring 5 × 20 mm. During the procedure under conscious sedation, brain protection with a filter (SPIDER RX^®^ 4 mm SPD2-040-320) was used. The control DSA at the end of the procedure revealed no residual stenosis (Figure 1F). During the procedure, the patient received 4000 IU of unfractionated heparin intravenously. The entire procedure was uneventful. During and immediately after the procedure, the patient had no focal neurological deficits.

**Figure 2:**
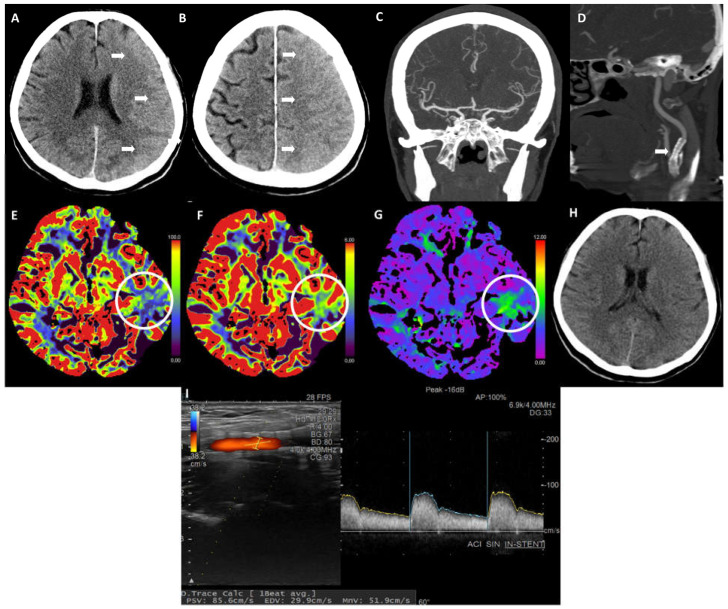
Neurological worsening. (**A**,**B**) A post-procedure CT showing sulcal effacement in the left cerebral hemisphere denoting edema (arrows). (**C**) A CTA post-procedure showing patent intracranial arteries. (**D**) A CTA post-procedure showing a patent stent in the left ICA (arrow). (**E**) A CT perfusion (CTP) post-procedure showing attenuated cerebral blood flow (CBF) in the left temporal region (circle). (**F**) The CTP shows attenuated cerebral blood volume (CBV) in the left temporal region (circle). (**G**) A CTP showing delayed TMAX in the left temporal region (circle). (**H**) A follow-up CT two days post-procedure denoting the improvement of the edema in the left hemisphere. (**I**) A follow-up DUS one month post-procedure showing a patent stent in the left ICA with normal hemodynamics.

One hour after the procedure, isolated motor dysphasia (NIHSS 3) was noticed by the staff. The BP values were low all the time intraprocedurally and after the procedure, i.e., 90–100/60–70 mmHg. There were no significant deviations in the laboratory blood tests. An immediate follow-up multimodal CT scan showed an edema of the major part of the left cerebral hemisphere, with a mildly hyperdense cortex (Figure 2A,B). A repeated CTA revealed a properly expanded stent in the left ICA and there were no visible arterial occlusions intracranially (Figure 2C,D). A CT perfusion (CTP) showed a small area in the left temporal region with a marginally lower cerebral blood flow (CBF), preserved cerebral blood volume (CBV), and mildly prolonged Tmax (Figure 2E–G). 

Due to cerebral edema, the patient received intravenous osmotic therapy with 20% mannitol (250 mL/8 h) until the next day. The speech disorder resolved within 30 min; the next day we did not notice any focal neurological signs (NIHSS 0).

The day after the procedure, the DUS showed an adequately patent stent in the left ICA. The flow velocities and sonograms were within the normal range; 0.50/0.12 m/s in the left common carotid artery (CCA), 1.07/0.31 m/s in the left ICA inside the stent, and 1.06/0.29 m/s distal to the stent. During the subsequent hospitalization, the patient was neurologically stable. A follow-up head CT scan after two days showed a mild edema of the left cerebral hemisphere (Figure 2H). 

A follow-up DUS after one month showed a properly expanded and patent stent in the left ICA with adequate hemodynamics (flow velocity in the ICA 0.86/0.29 m/s) (Figure 2I). The patient did not report any new neurological symptoms nor any speech difficulties. She had been receiving the same pharmacotherapy as at the discharge, namely aspirin, clopidogrel, rosuvastatin, proton pump inhibitor, and previous COPD therapy.

## 3. Literature Review

In total, 65 studies were deemed relevant to the topic of the current paper. The studies included were deemed to be of high quality, as indicated by the high Newcastle-Ottawa total score (median = 8 [6,7,8,9]), and both pairs of raters exhibited statistically significant agreement (*K* = 0.935, *z* = 8.05, *p* < 0.001 and *K* = 0.33, *z* = 2.85, *p* = 0.004).

### 3.1. Prevalence of CHS

The prevalence of CHS after the carotid revascularization was reported in 44 studies (Table 1). The prevalence of CHS after CAS was reported in 24 studies [1,4,6,7,8,9,10,11,12,13,14,15,16,17,18,19,20,21,22,23,24,25,26,27] and the weighted average was 1.2% [0.0–37.7%]. The prevalence of CHS after CEA was reported in 19 studies [1,8,21,22,28,29,30,31,32,33,34,35,36,37,38,39,40,41,42] and the weighted average was 0.29% [0.15–20%]. The prevalence of intracranial hemorrhage (ICH) after CAS was reported in 15 studies [4,7,9,10,11,12,15,16,20,26,27,43,44,45,46] and the weighted average was 0.51% [0–9.3%]. The prevalence of ICH after CEA was reported in 6 studies [16,39,43,44,47,48], and the weighted average was 0.07% [0.02–0.6%].

### 3.2. Diagnosing and Predicting CHS

A total of 19 manuscripts explored various imaging techniques used to diagnose and predict CHS [17,18,24,32,33,35,37,42,49,50,51,52,53,54,55,56,57,58,59], DUS [33,35,42,51,57], DSA [24], CTP [59], single-photon emission computed tomography (SPECT) [17,37], functional near-infrared spectroscopy (fNIRS) [18,54,57], glucose cerebral oxygen extraction fraction [53,56], and magnetic resonance imaging (MRI) techniques, such as arterial spin labeling (ASL) [32,49,52,55,58] and perfusion-weighted MRI [50] can be used to diagnose and predict CHS. Regarding the specific MRI techniques, multidelay ASL was more accurate than single post-labeling delay ASL [32,52,55]; however single post-labeling delay can be useful if combined with MR angiography [49] to diagnose CHS accurately. Interestingly, a significant correlation was found between the preoperative cerebral blood volume and increases in cerebral blood flow immediately after CEA with elevated preoperative CBV being the only significant independent predictor of post-CEA hyperperfusion in a perfusion-weighted MRI study [58]. A direct comparison between the methods is lacking.

### 3.3. Risk Factors for CHS

A total of 17 manuscripts dealt with risk factors predisposing patients to CHS [1,6,11,12,27,28,30,34,36,39,40,48,60,61,62,63,64]. Clinical and morphological features, such as a recent (<1 month) history of transient ischemic attack or stroke [36,39], recent (<3 months) contralateral CEA [40], urgent intervention [1], acute carotid occlusion [1], contralateral ≥70% stenosis [36], and the presence of leptomeningeal collaterals (LMC) [11] were associated with CHS. Furthermore, a need for postoperative intravenous BP medication [1] and diabetes mellitus [27] were associated with the development of CHS. Patients experiencing prolonged hemodynamic instability after CAS, characterized by hypertension, hypotension, and/or bradycardia, were found to be at a higher risk for CHS [6,12,28,30,34,36,48,60,61,62,63,64]. However, none of the articles mentioned isolated hypotension as a risk factor for CHS. Age was identified as a risk factor in a smaller study [17] but was not confirmed subsequently [1].

### 3.4. Prevention of CHS

Prevention of CHS was the subject of 8 articles [1,15,20,26,45,63,65,66]. In 2 articles, adequate post-stenting BP management was found to be efficient in high-risk patients [15,63]. Angiotensin-converting enzyme inhibitors and angiotensin II receptor blockers were found to decrease the risk for postoperative CHS [1]. One study has shown that strict postoperative BP control is important for CEA and not CAS to prevent CHS [16]. Pretreatment with edaravone, a free radical scavenger, showed promising results in preventing the occurrence of CHS after CEA [66]. Staged angioplasty, a two-stage form of CAS, is effective in preventing CHS [45,65]. The gentle carotid artery stent placement strategy, which involves intentional residual stent stenosis, may prevent CHS in high-risk patients [20].

### 3.5. Prognosis of CHS

Eleven articles were identified regarding the prognosis of CHS [4,9,10,11,15,26,36,44,46,47,67]. Cerebral hyperperfusion, even when asymptomatic, may result in cognitive impairment without visible structural brain damage on MRI [67]. CHS post-CAS carried a 0.7 [4] to 1.43% [10] rate of ICH development. Mortality after ICH ranged from 40 to 75% for CAS [9,15,26,44,46] and 38 to 58% for CEA [36,44,47]. One study compared the in-hospital mortality after CAS and CEA and they found a significantly higher mortality after CAS (1.1% vs. 0.6%, respectively) [43].

## 4. Discussion

Our paper introduces a case report that diverges from the results of a comprehensive meta-analysis on CHS following CAS, which provides valuable insights into CHS. Notably, our patient demonstrated a distinct profile characterized by: (1) the absence of arterial hypertension; (2) hypotension during and after the procedure; and (3) only moderate but symptomatic carotid stenosis at the time of intervention. These factors may offer new insights into the variability of CHS presentations post-CAS. To the best of our knowledge, this is the first case report of a hypotensive patient with CHS. We provide similarities and differences between our case and the published data after conducting a thorough literature review. 

What may be unique in our case is the fact that the patient exhibited only a few risk factors associated with the development of CHS, such as the female sex and potentially symptomatic stenosis [6,12,28,30,34,36,48,60,61,62,63,64], which at the time of the procedure turned out to be only moderate. Post-procedural hypotension due to carotid baroreceptor overstimulation is a frequently encountered phenomenon after CAS, which was also present in our case. According to the literature review, no article has mentioned isolated post-CAS hypotension as a risk factor for CHS. Instead, post-procedural hypotension could be regarded as indicative of a possible hemodynamic instability [60], contributing to the development of CHS, with an interplay of other risk factors. 

Conversely, our patient did not exhibit other established risk factors for CHS, such as an isolated hemisphere indicating inadequate primary collateral vessels (e.g., an incomplete circle of Willis), nor a high-grade carotid stenosis, particularly in the absence of high-grade contralateral stenosis [31]. In a study involving 455 patients, only 3 out of 9 CHS cases had systolic BP exceeding 160 mmHg at the onset of CHS symptoms [40]. Additionally, the severity of ipsilateral and contralateral ICA stenoses did not significantly differ between CHS cases and the remaining cases [40]. These findings appear to contradict the prevailing notion that CHS predominantly arises in patients with severe ipsilateral or contralateral carotid disease or severe hypertension, a perspective consistent with our case.

A vast array of different diagnostic radiological and/or functional modalities before carotid revascularization procedures trying to predict CHS with reasonable reliability have been utilized in studies [15,17,18,24,29,32,33,35,40,42,49,50,51,52,53,54,55,56,57,58,59]. A preoperative cerebrovascular reserve assessment utilizing TCD [42], and advanced cerebral perfusion imaging, such as MRI-based arterial spin labeling [68] may aid in CHS risk stratification. In turn, identifying these high-risk patients in advance may potentially enable the prevention of CHS. 

Per our institution’s protocol, elective CAS or CEA patients are not required to undergo additional sophisticated investigations (e.g., SPECT) before carotid revascularization. A non-contrast brain CT scan and an aortocervical and intracranial CTA combined with carotid DUS are mandatory at our institution, which had all been performed on our patient prior to CAS. Summing up these investigations, there were no apparent radiological risk factors associated with the development of CHS in our patient prior to CAS apart from a small postischemic encephalomalacia region in the territory of the left middle cerebral artery. The stent used in our patient was a typical carotid artery bare metal stent and a brain protection device with a filter was used during the procedure. However, the grade of carotid stenosis differed significantly between pre-CAS CTA and intraprocedural DSA in our patient. This difference may be partly ascribed to different diagnostic modalities (CTA vs. DSA), but based on the magnitude of the difference (90% vs. 50%), we suspect that the stenosis grade must have truly diminished due to pleiotropic effects of a potent statin therapy she had been receiving at least 6 months before the procedure [69,70]. This is biologically plausible and not infrequently seen in lipid-rich carotid plaques in daily clinical practice. 

Upon the occurrence of motor dysphasia post-CAS in our patient, we performed multimodal CT imaging as per our institution’s protocol. A non-contrast CT showed signs of cerebral vasogenic edema affecting the ipsilateral cerebral hemisphere, the CTA showed a patent stent, and there were no signs of an intracranial arterial occlusion. Curiously, the CTP revealed only a small area of a prolonged TTP with mildly attenuated CBF and preserved CBV in the left temporal region. Indeed, there were no apparent signs of CTP associated with cerebral hyperperfusion, such as enhanced CBF and/or CBV and/or shortened circulation times in our patient. 

The symptoms of CHS in our patient emerged within the first hour following the completion of the revascularization procedure, consistent with the findings from published studies on CHS after CAS [16]. Conversely, after CEA, CHS typically manifests over the ensuing days [58]. The patient was treated with an intravenous osmotic diuretic mannitol, usually administered to relieve vasogenic edema, and she fully recovered within a few days clinically. A follow-up CT showed a substantial improvement in the cerebral edema. 

Carotid endarterectomy and CAS share most of the risk factors for CHS, such as arterial hypertension, older age, contralateral ≥70% carotid stenosis, and recently symptomatic stenosis). Interestingly, a meta-analysis including more than 236,000 procedures (18,393 CAS) could not calculate potential differences in risk factors for CHS after CEA compared to CAS due to inadequate pooled data [71]. Nevertheless, there may be differences in risk factors for CHS between the two techniques. These primarily stem from the technical reasons (general anesthesia (CEA) vs. local anesthesia (CAS), iodinated contrast medium (in CAS), the “typical” patient profile (possibly more fragile patients in CAS), and stricter medication protocol in patients undergoing CAS (double antiplatelets). Carotid stenting is more frequently associated with prolonged hemodynamic instability, which may be regarded as a more significant risk factor for CHS in CAS compared to CEA patients [6,12,28,30,34,36,48,60,61,62,63,64]. 

According to our analysis, CHS seems to be more prevalent following CAS compared to CEA. In CAS, a more sudden restoration of a high blood flow compared to a more gradual reestablishment of blood flow in CEA, may lead to a rapid increase in cerebral perfusion pressure and a higher likelihood of CHS. Patients undergoing CAS may experience more significant fluctuations in blood pressure during the procedure, which can increase the risk for CHS. The abrupt changes in hemodynamics seen with CAS can affect the brain’s ability to autoregulate cerebral blood flow, further exacerbating the risk of CHS. Carotid stenting is often performed in patients who may be considered high-risk surgical candidates (older, frail patients or those with multiple comorbidities). This population may already have impaired cerebral autoregulation or other risk factors that make CHS more likely after CAS. The design and deployment technique of the stent can also influence blood flow patterns after the procedure. There may be variations in how well stents support flow dynamics compared to the physiological flow restoration that occurs during CEA. The types of lesions being treated can differ between patients undergoing CAS (predominantly lipid-rich plaques with a higher potential of embolization) and those undergoing CEA (stable calcified plaques), potentially affecting thromboembolic rates and subsequent cerebral perfusion changes. The differences in postoperative monitoring and management of blood pressure and cerebral perfusion between the two procedures can also influence the incidence of CHS. 

Our systematic analysis produced noteworthy results that partially diverge from the comprehensive meta-analysis conducted by [5]. Contrary to their findings, we have found a lower prevalence of CHS after CAS. What is more, a symptomatic status is a significant risk factor for CHS according to our results, contrasting with the aforementioned meta-analysis [5].

CHS is a rare potentially harmful complication in patients undergoing revascularization procedures of both extracranial and intracranial arteries [1,4,6,7,8,9,10,11,12,13,14,15,16,17,18,19,20,21,22,23,24,25,26,27,28,29,30,31,32,33,34,35,36,38,39,40,41,42,43,44,45,46,47,48]. Its most feared consequence, though relatively infrequent, is ICH, which can be fatal in up to 75% [4,9,10,11,15,20,26,36,44,46,47]. However, since mortality was not reported in all studies, the numbers could be overestimated. While stringent control of postoperative BP effectively prevents ICH in patients with CHS following CEA, there seems to be no clear correlation between BP management and ICH in those experiencing CHS after CAS [16]. We posit that uninterrupted dual antiplatelet therapy and intraprocedural heparinization during CAS may contribute to the risk of ICH associated with CHS in post-CAS patients, which is approximately seven-fold higher than that after CEA [4,7,9,10,11,12,15,16,20,26,27,39,43,44,45,46,47,48]. According to the existing literature, the presence of LMC represents a significant risk factor for ICH following CAS [11]. The prominence of these vessels on the CTA suggests inadequate primary collateral circulation. Our patient, who did not experience ICH, did not exhibit clearly visible LMC on the pre-CAS CTA scan and the left PCoA was clearly visible. However, CHS without ICH carries a much better prognosis, with the majority of patients recovering completely [12], which is in line with our case. However, cerebral hyperperfusion may be associated with post-procedural cognitive impairment [67], which has not been dealt with in our patient and is generally mostly neglected in these patients in daily clinical practice. 

According to the presented case, CHS can develop even in the absence of AH, notably, in hypotensive patients. Here, the situation becomes more complex. In general, hypotension further reduces the cerebral blood flow, which might additionally compromise the brain’s adaptation to post-stenting hyperperfusion. Hypotension exacerbates this by reducing perfusion pressure, leading to fluctuations between hyperperfusion and hypoperfusion, further hindering cerebral autoregulation [2]. In case the myogenic component of autoregulation fails, the remaining autoregulation depends on the innervation of the sympathetic autonomic system [2]. Complicating matters further, hypotension may indeed point to the preexisting autonomic dysfunction elevating the risk of CHS [72]. In a chronically ischemic brain (e.g., extensive leukoaraiosis), the arterioles and capillaries are vulnerable to rupture and bleeding when perfusion pressure abruptly increases, predisposing these patients to CHS and even ICH. 

In our opinion, endothelial dysfunction resulting from chronic ischemia due to carotid stenosis may serve as the initial mechanism, triggering the cascade leading to CHS. Prolonged hypoperfusion in conjunction with endothelial dysfunction, may compromise the integrity of the BBB, which is a prerequisite for vasogenic brain edema as one possible manifestation of CHS, seen in our patient as well. Furthermore, reperfusion can incite an inflammatory response, exacerbating vascular permeability and additionally exacerbating CHS. A presumed mediator of impairment of autoregulation and dysfunction of BBB in CHS is nitric oxide (NO), which leads to vasodilatation and increases in cerebral vessel permeability [73]. Reactive oxygen species damage the cerebrovascular endothelium, further exacerbating postoperative hyperperfusion. The breakdown of carotid body baroreceptors in either CEA or CAS may damage the ability to respond to acute changes in systemic arterial BP [74]. An interesting and less-mentioned hypothesis involves the trigeminovascular reflex. Following exposure to vasoconstrictors, the trigeminovascular system releases vasoactive neuropeptides, resulting in increased cerebral blood flow, to return vascular tone to the baseline [2].

The management of hypotensive patients undergoing CAS can be challenging. The blood pressure should be monitored continuously via an arterial line or checked every 15 min, during, and at least 24 hours after the procedure. Usual treatments of CAS-related hemodynamic depression (either hypotension and/or bradycardia) include intravenous fluid resuscitation and intravenous inotropes such as dopamine and norepinephrine [75]. Additionally, patients receive atropine during the procedure. Usually, these treatments are effective, but in case of an unfavorable response, other treatments such as oral midodrine have been suggested [76]. It is prudent to cautiously monitor blood pressure for at least 1 month for the restoration of cerebral autoregulation [2].

Our case, in conjunction with the existing literature, indicates a complex interplay of factors influencing the pathophysiology of CHS, which is at present not fully understood. This underscores the need for additional research to refine diagnostic and management strategies for this condition.

## 5. Materials and Methods

**Figure 3:**
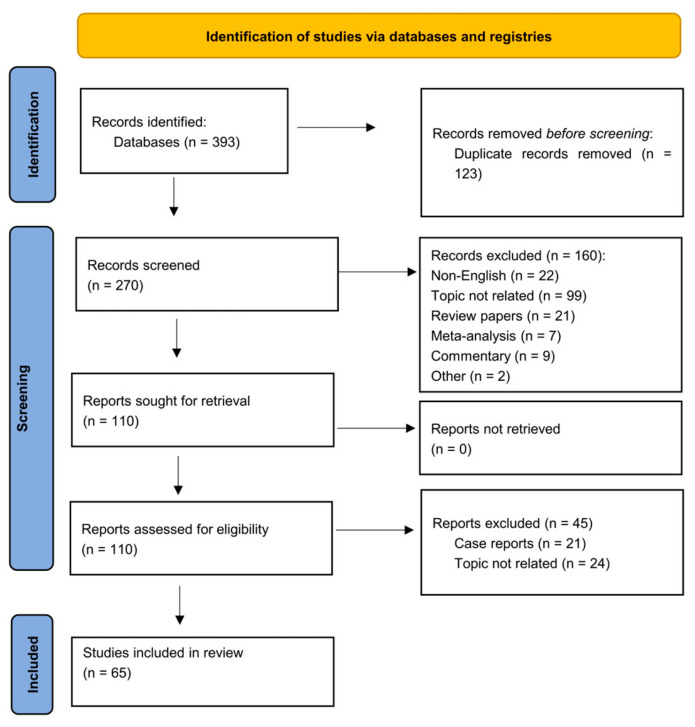
Flowchart of the selection procedure according to the PRISMA2020 guidelines.

A literature search on PubMed was performed using the terms »cerebral hyperperfusion syndrome«, »hypotension«, »hyperperfusion«, »stroke«, »intracranial hemorrhages«, »risk factors«, »carotid revascularization«, »carotid stenting«, »carotid endarterectomy«, »blood-brain barrier«, »endothelium«, and »contrast encephalopathy«. The search involved a combination of the aforementioned terms (the full list of search strings is available in Supplementary Materials) on 31 January 2024. A total of 393 records were identified and after the removal of 123 duplicates, we screened the remaining 270 abstracts. The first 135 abstracts were screened, and relevant full papers were evaluated by M.Z. and S.F., while the second 135 were screened and evaluated by M.P. and S.F. In case of disagreement, the third screener (either M.Z. or M.P.) broke the tie. The study quality was assessed using the Newcastle-Ottawa Quality Assessment Scale. Only the original research papers relevant to the rationale of the present paper, written in English were included in the review. A total of 115 full texts were screened and after the exclusion of 21 case reports or case series and 24 papers with unrelated topics, the remaining 65 studies were included in the systematic literature review. The identified papers were categorized into one or more of the following categories: prevalence, diagnosing and predicting, risk factors, prevention, and prognosis. The selection procedure is shown in Figure 3.

## 6. Conclusions

Here, we report a patient without AH who developed CHS post-CAS for symptomatic moderate carotid stenosis while being hypotensive. To the best of our knowledge, this is the first case report of a hypotensive patient with CHS. Our patient’s case highlights the complexity of CHS, involving factors like endothelial dysfunction and sudden reperfusion. Further research is needed to refine diagnostic and management approaches for this condition.

## Figures and Tables

**Table 1:** Studies reporting on CHS prevalence.

Authors	Year	CAS	CEA	N of CHS	N of ICH	Total N	Prevalence of CHS (%)	Prevalence of ICH (%)
Hsu et al. [1]	2023	x	x	329		156,003	0.21 (CAS: 0.53, CEA: 0.15)	
Timaran et al. [43]	2009	x	x		39	135,903		0.029 (CAS: 0.14, CEA: 0.016)
Wang et al. [36]	2017		x	94		51,001	0.18	
Hussain et al. [44]	2018	x	x		80	16,688		0.48 (CAS: 0.86, CEA: 0.42)
Ogasawara et al. [16]	2007	x	x	61	27	4494	1.4 (CAS: 1.1, CEA: 1.9)	0.60 (CAS: 0.72, CEA: 0.38)
Henderson et al. [47]	2001		x		12	2747		0.44
Huibers et al. [21]	2015	x	x	9		1713	0.53 (CAS: 0.38, CEA: 0.73)	
Ziaja et al. [22]	2014	x	x	127		1386	9.2 (CAS: 8.0, CEA: 10.6)	
Maas et al. [39]	2013		x	14	4	841	1.7	0.48
González García et al. [4]	2019	x		22	5	757	2.9	0.66
Lee et al. [48]	2022		x		1	735		0.1
Brantley et al. [25]	2009	x		7		482	1.45	
Ascher et al. [40]	2003		x	9		455	2.0	
Abou-Chebl et al. [26]	2004	x		5	3	450	1.1	0.67
Wang et al. [27]	2017	x		17	3	382	4.5	0.79
Lee et al. [11]	2016	x		4	4	228	1.8	1.75
Yang et al. [7]	2016	x		2	6	224	0.9	2.68
Lai et al. [41]	2015		x	6		185	3.2	
Pennekamp et al. [42]	2012		x	10		184	5.4	
Tan et al. [9]	2009	x		4	2	170	2.4	1.18
Xu et al. [45]	2022	x			13	153		8.5
Li et al. [6]	2016	x		55		146	37.7	
Meyers et al. [10]	2000	x		7	2	140	5.0	1.43
Narita et al. [24]	2013	x		3		136	2.2	
Ogawa Ito et al. [23]	2019	x		11		125	8.8	
Fan et al. [38]	2021		x	10		124	8.1	
Hayashi et al. [8]	2012	x	x	2		101	2.0 (CAS: 2.1, CEA: 1.9)	
Maltezos et al. [28]	2007		x	14		100	14.0	
Manojlovic et al. [30]	2020		x	18		93	19.4	
Morrish et al. [46]	2000	x			4	90		4.44
Li et al. [33]	2024		x	18		90	20.0	
Ogasawara et al. [34]	2008		x	11		80	13.8	
Fan et al. [32]	2023		x	15		79	19.0	
Ogasawara et al. [35]	2005		x	2		67	3.0	
Katano et al. [31]	2012		x	4		65	6.2	
Matsumoto et al. [18]	2009	x		2		64	3.1	
Henry et al. [12]	2005	x		2	1	57	3.5	1.75
Tseng et al. [14]	2009	x		3		55	5.5	
Chang et al. [15]	2011	x		11	5	54	20.4	9.26
Choi et al. [19]	2010	x		4		48	8.3	
Hoffmann-Wieker et al. [29]	2022		x	2		45	4.4	
Kaku et al. [17]	2004	x		3		30	10.0	
Mori et al. [20]	2021	x		0	0	28	0.0	0.00
Son et al. [13]	2015	x		1		22	4.5	

CAS–carotid artery stenting, CEA–carotid endarterectomy, N–number, CHS–cerebral hyperperfusion syndrome, ICH–intracerebral hemorrhage. X denotes whether a study included patients after CAS, CEA, or both groups.

## Data Availability

All the data generated or analyzed during the study are included in this article. Further inquiries can be directed to the corresponding author.

## Supplementary Materials

The following supporting information can be downloaded at: https://www.mdpi.com/article/10.3390/life14111472/s1, Textfile 1: Search strings used for systematic review.
